# The Effect of Inactivated Mycobacterium Paratuberculosis Vaccine on the Response to a Heterologous Bacterial Challenge in Pigs

**DOI:** 10.3389/fimmu.2019.01557

**Published:** 2019-07-05

**Authors:** Kristoffer Jarlov Jensen, Mette Sif Hansen, Peter Mikael Helweg Heegaard, Christine Stabell Benn, Gregers Jungersen

**Affiliations:** ^1^Bandim Health Project, University of Southern Denmark, Copenhagen, Denmark; ^2^Department of Health Technology, Technical University of Denmark, Lyngby, Denmark; ^3^National Veterinary Institute, Technical University of Denmark, Lyngby, Denmark; ^4^Department of Biotechnology and Bioengineering, Technical University of Denmark, Lyngby, Denmark; ^5^OPEN, Institute of Clinical Research, University of Southern Denmark, Odense, Denmark

**Keywords:** non-specific effects of vaccines, heterologous immunity, paratuberculosis vaccine, actinobacillus pleuropneumoniae, pigs (sus domesticus), mycobacterial vaccine

## Abstract

**Background:** Vaccines may have non-specific effects, affecting resistance to heterologous pathogens. Veterinary vaccines have seldom been investigated for their non-specific effects. However, recent observational studies suggest that an inactivated paratuberculosis vaccine reduced all-cause mortality in goats and cattle.

**Aim:** We tested if vaccination with a killed mycobacterial vaccine may have heterologous effects in swine (Sus domesticus), specifically on the pathogenic and clinical effects of a heterologous challenge with Actinobacillus pleuropneumoniae in young pigs.

**Methods:** Newborn piglets were randomized to vaccination s.c. with the inactivated paratuberculosis vaccine Gudair (Zoetis Inc.) (*n* = 17) or no vaccine (*n* = 16). At 4–5 weeks after vaccination, all piglets were challenged intra-nasally with a high (Gudair: *n* = 8; control: *n* = 8) or a low (Gudair: *n* = 9; control: *n* = 8) dose of the gram-negative bacterium A. pleuropneumoniae causing acute porcine pleuropneumonia. The effect and severity of pathogen challenge was evaluated by measuring acute phase proteins C-reactive protein, haptoglobin and Porcine α1-acid glycoprotein, and by gross pathology 1 day post challenge. Specific and non-specific *in vitro* cytokine responses to vaccination were evaluated in whole blood before bacterial challenge.

**Results:** The vaccine was immunogenic in the pigs as evidenced by increased IFN-γ responses to purified protein derivative of Mycobacterium paratuberculosis. However, Gudair vaccine did not affect IL-6 responses. The gross pathology of the lungs as well as the acute phase protein responses after the high A. pleuropneumoniae dose challenge was slightly increased in the vaccinated animals compared with controls, whereas this was not seen in the animals receiving the low-dose bacterial challenge.

**Conclusion:** The inactivated paratuberculosis vaccine exacerbated the pathological and inflammatory effects of an experimental A. pleuropneumoniae infection in young pigs.

## Introduction

Observational studies and randomized controlled trials in humans have shown that many childhood vaccines can affect resistance to other infections than the targeted disease; a phenomenon called non-specific effects of vaccines or heterologous immunity (1). The non-specific effects may be beneficial, thereby decreasing susceptibility to other infections, or they may be detrimental, i.e., increasing susceptibility to other diseases. Common human vaccines with beneficial non-specific effects include the live vaccines bacillus Calmette-Guérin (BCG) against tuberculosis (2), measles vaccine (3) and oral polio vaccine (4). In contrast, non-live vaccines like the inactivated diphtheria-pertussis-tetanus vaccine may have negative non-specific effects (5). These non-specific effects of vaccines, beneficial as well as negative, have generally been most pronounced in females (3, 5).

The biological mechanisms behind the non-specific effects of vaccines are not known, and may comprise different immunological mechanisms, depending on the vaccine in question. For BCG, one potential mechanism is trained innate immunity, in which the innate immune system acquires an enhanced ability to respond to heterologous innate stimulation, mediated by epigenetic reprogramming of monocytes (6). Although the evidence from epidemiological and immunological studies in humans has been corroborated by experimental animal models, particularly in mice (7), very few studies have investigated potential non-specific effects of veterinary vaccines, despite the fact that veterinary vaccines are administered routinely in virtually all commercial animal production systems to an increasing number of animals. If veterinary vaccines have non-specific effects, there may be potential to optimize the current use of vaccines to production animals, thereby reducing mortality and morbidity, antibiotics use and the implicated financial losses.

Recently, it was reported that goats allocated to the commercial heat-inactivated oil-in-water emulsified Gudair vaccination (Zoetis Inc.) against *Mycobacterium avium* subsp. *paratuberculosis* infection had a significant reduction in all-cause culling compared to non-vaccinated peers, regardless of age. Pathological examinations of the culled corpses detected lesions consistent with paratuberculosis infection only in adults, indicating that Gudair vaccine conferred protection against non-paratuberculosis infection (8). A similar non-specific beneficial effect has also recently been reported in cattle for which the bovine version of killed paratuberculosis vaccine (Silirum, Zoetis Inc.) given before age 3 months was associated with a reduction in all-cause culling to an extent, which seemingly exceeded the specific protection against paratuberculosis, as indicated by post-mortem examination of slaughtered cows in similar herds (9).

Gudair vaccine contains inactivated heat-killed whole *M. avium* subsp. *paratuberculosis* of the *M. tuberculosis* complex, which also includes *M. tuberculosis* and *M. bovis* BCG. Beneficial non-specific effects of heat-killed *M. tuberculosis* in the oil-emulsified formulation known as Freund's complete adjuvant (FCA) were demonstrated several decades ago. E.g., mice pre-treated with FCA had markedly reduced viremia following inoculation with Foot-and-mouth disease virus (10). Recently, a commercial veterinary vaccine containing cell wall components from *M. phlei* in oil-emulsion was reported to decrease all-cause morbidity in feedlot cattle (11), and to improve survival after Enterotoxigenic *Escherichia coli* induced diarrhea in neonatal cattle (12). Early murine experimental studies of non-specific effects also found that heat-killed BCG or *M. fortuitum* administered s.c. or i.p. improved survival after subsequent *Staphylococcus aureus* infection alone (13) or *S. aureus* mixed with endotoxin (14), and crude cell wall extract from *M. phlei* administered i.p. or i.v. improved survival after subsequent infections with *S. aureus* or *Salmonella enteritidis* (15). The mortality reducing effect of heat-killed BCG persisted from 13 days through 10 weeks after immunization (13).

Although the above studies using paratuberculosis vaccine did take the vaccine-specific protection against paratuberculosis into account, it would be desirable to reproduce these data in paratuberculosis-free settings. Previously, paratuberculosis vaccination of goats (16) and cattle (17) was shown to reduce gross pathology and bacterial colonization after experimental exposure to *M. caprae* and *M. bovis*, respectively, more than 3 months after immunization. This heterologous protection offered by *M. paratuberculosis* vaccination to other mycobacterial infections could be due to cross-reactive T-cell mediated adaptive immunity or innate training-like mechanisms.

As paratuberculosis is not endemic to slaughter pigs, no such vaccine is licensed to use in pigs. This may reduce the risk of adaptive cross-reactive mechanisms playing any significant role in the study, making the pig a relevant model to investigate if the non-specific protection is mediated by innate immune mechanisms in addition to the relatively high physiological similarity between pigs and humans (18).

We aimed to investigate if vaccination of piglets with the inactivated paratuberculosis vaccine Gudair may non-specifically influence the pathology and inflammation following a subsequent challenge with the Gram-negative, facultative anaerobic coccobacillus *Actinobacillus pleuropneumoniae* causing acute porcine pleuropneumonia.

## Materials and Methods

Three weeks prior to farrowing, three pregnant sows (Danish Landrace/Danish Yorkshire crossbreeds) were immunized sub-cutaneously behind the left ear base with 1 ml Gudair (heat-inactivated *M. avium* subsp. *paratuberculosis* F316 strain, lot # 160209, CZ Veterinaria).

### Randomization

**Figure 1:**
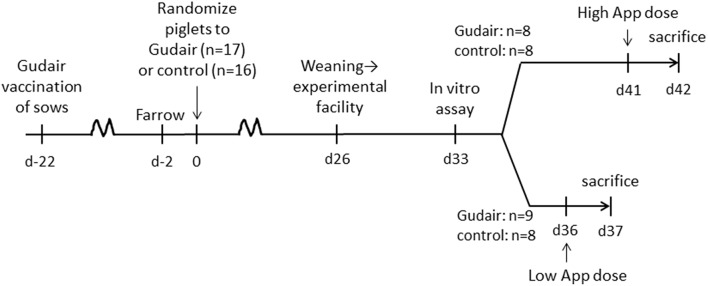
Timeline of experiment.

Within 2 days after farrowing, the three litters of piglets (Danish Landrace/Danish Yorkshire crossbreeds, paternal lineage Duroc, *n* = 33) were allocated by litter alternatingly to receiving either Gudair vaccine (*n* = 17, 0.5 ml, s.c. in the right shoulder) or nothing (*n* = 16, control group) (Figure 1). The piglets were tailed docked, but males were not castrated.

### Follow-Up Post-randomization

After weaning, the piglets were transferred to the experimental facilities at the Technical University of Denmark, and placed in two boxes with a balanced distribution of vaccination treatment, sex, weight and litter, and to intranasal challenge with *A. pleuropneumoniae* scheduled on day 36 or 41 after vaccination, respectively.

### Challenge

A seed lot of *A. pleuropneumoniae* Danish field strain 4226, serotype 2 (19) stored at −80°C was cultured overnight at 37°C on modified pleuropneumonia-like organism (PPLO)-agar plates using *E. coli* as a nurse strain, suspended in 0.9% NaCl, and adjusted to desired concentration by turbidity. Pigs were lightly anesthetized with (Zolazepam/Tiletamine) and inoculated by spraying the bacterial solution with a manual nebulizer (LMA Mad Nasal, Teleflex) directly into the nostrils.

#### Low-Dose Challenge

At 36 days after vaccination, pigs in the low-dose group received 1.5 × 10^8^ CFU *A. pleuropneumoniae*/animal administered by 1 ml in either nostril.

#### High-Dose Challenge

At 41 days after vaccination, pigs in the high-dose group received 1 × 10^9^ CFU *A. pleuropneumoniae*/animal administered by 1 ml in either nostril.

Concentration and purity of the inoculation suspensions were verified by seeding on PPLO and blood agar plates, respectively.

### Follow-up Post-challenge

Within 24 h of *A. pleuropneumoniae* inoculation, animals were sedated by Zoletil (Zolazepam/Tiletamine) and sacrificed by captive bolt pistol and bleeding.

Necropsy was performed for characterization of gross lesions. The injection site was examined and lung lesions were scored semi-quantitatively in regard to severity modified after Baarsch et al. (20); 0 = no lesions, 1 = hemorrhage, non-consolidated processes; 2 = small localized *A. pleuropneumoniae*-like lesions; 3 = large, extensive AP-like lesions. *A. pleuropneumoniae*-like lesions were defined as areas with hemorrhage, lung consolidation and necrosis (red hepatization), and fibrin exudation.

For re-isolation of the inoculation strain from lung (Lobus cranialis dexter), liver (Lobus hepatis sinister lateralis) and spleen a scalpel was inserted into the tissue and struck on PPLO agar and cultured at 37°C; the following day, emerging colonies were enumerated and categorically rated as 0, 1–10, 11–50, 51–100, or >100 colonies.

Weight and rectal temperatures were evaluated before and after challenge.

### Blood Samples

On day 33 after vaccination (before challenge), heparinized blood was collected from the jugular vein, distributed as 1 ml/well in 24-well flat bottom plates (Costar) for overnight (23 h) incubation in humidified 37°C, 5% CO_2_ atmosphere with the following stimulation panel (final concentrations in cultures are indicated):

Purified protein derivative of Johne's disease (PPDj, 10 ug/ml, Promise strain, DTU National Veterinary Institute), lipopolysaccharide from *E.coli* (LPS, 10 ng/ml, Sigma), Pam3CSK4 (1 ug/ml, Invitrogen), PHA-L (2 ug/ml, Sigma) or medium alone. Supernatants were harvested and stored at −20°C until quantification by monoclonal sandwich ELISAs of IL-6 (Porcine IL-6 DuoSet ELISA, R&D Systems) or IFN-γ (21).

#### Acute Phase Protein ELISAs

C-reactive protein (CRP), haptoglobin and porcine α1-acid glycoprotein (PAGP) were analyzed from serum samples collected on day 0 and day 1 of *A. pleuropneumoniae* inoculation using in-house ELISA protocols (22–24).

### Statistics

Data was analyzed in StataMP ver. 12 (StataCorp, US) or GraphPad Prism ver6 (GraphPad Software) using Kruskal-Wallis test for IFN-γ, IL-6, CRP, haptoglobin, weight, temperature and colony count data in unpaired analysis and using Wilcoxon matched-pairs signed-rank test for paired analyses. For haptoglobin, IL-6 and IFN-γ, a few observations were above or below the assay range and were assigned the value of the highest or lowest, respectively, reliably quantified value of the standard curve in the statistical analysis. Pig α1-acid glycoprotein (PAGP) levels were analyzed as relative values to the index defined by the mean level of the low *A. pleuropneumoniae* dose control animals before challenge. Pathology scores were dichotomized as ≥2 vs. <2 and analyzed using Wilcoxon rank-sum test.

### Ethics

The animal experiments were approved by the Danish Animal Experiment Inspectorate, approval number 2015-15-0201-00520.

## Results

The piglets in the paratuberculosis vaccine and control groups were similar with respect to weight on the day of treatment allocation. There was no difference in the weight gain by vaccination status (Table 1).

**Table 1:** Background information.

	**Gudair**	**Control**
*N*	17	16
Sex male/female	8/9	11/5
Weight kg day0, median (min-max)	1.5 (1.1–2.0)	1.5 (1.1–2.4)
Weight kg day33, median (min-max)	8.0 (3.5–10.0)	8.3 (3.0–11.0)
App low dose / high dose	9/8	8/8

*App, Actinobacillus pleuropneumoniae*.

The paratuberculosis vaccinated sows showed no severe reaction after paratuberculosis vaccination. All paratuberculosis vaccinated piglets had a local reaction at the injection site, although the severity was variable, ranging from a diffuse cutaneous reaction with slight pus exudation to ulceration of the epidermis (Supplementary Figure 1).

**Figure 2:**
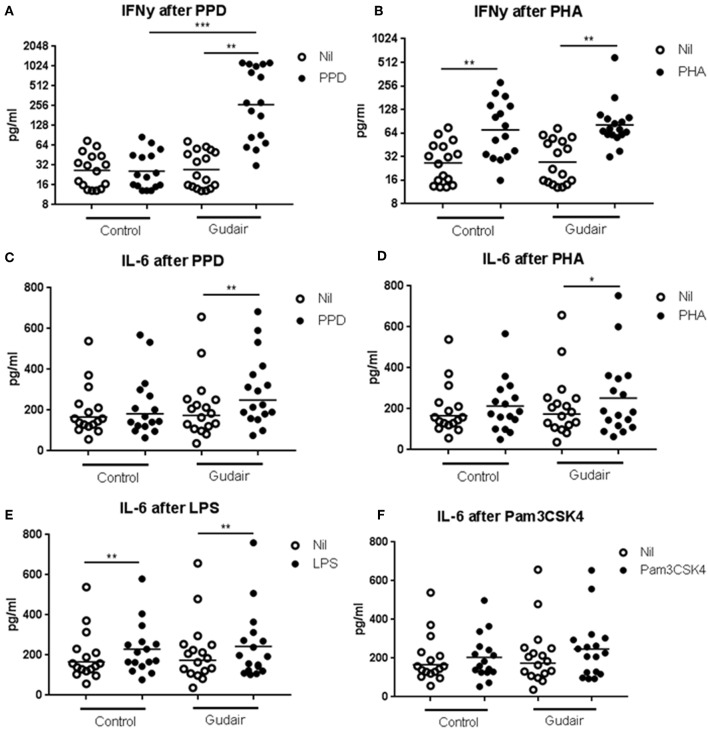
*In vitro* cytokine responses. Concentrations of cytokines of IFN-γ **(A,B)** and IL-6 **(C–F)** in whole blood cultures after overnight stimulation with purified protein derivative of *M. avium* subsp. paratuberculosis **(A,C)**, phytohaemagglutinin **(B,D)**, lipopolysaccharide **(E)** or Pam3CSK4 **(F)**, including the non-stimulated medium alone (Nil), comparing Gudair vaccinated (*n* = 17) with non-vaccinated control animals (*n* = 16). Statistical test for difference by vaccination using Kruskal-Wallis and for paired analysis of stimulation effect using Wilcoxon matched-pairs signed-rank test; *p < 0.05; **p < 0.01; ***p < 0.001. Note the different scales in the sub-graphs. Means of the subgroups are indicated on the graphs.

*In vitro* IFN-γ responses to PPDj were increased 33 days after paratuberculosis vaccination compared to the control group (Figure 2). There was no evidence of paratuberculosis vaccination inducing a differential IFN-γ response to the mitogen PHA or of a differential IL-6 response to PPDj or PHA, or to the innate TLR stimulants LPS (TLR4) or Pam3CSK4 (TLR2/1).

**Figure 3:**
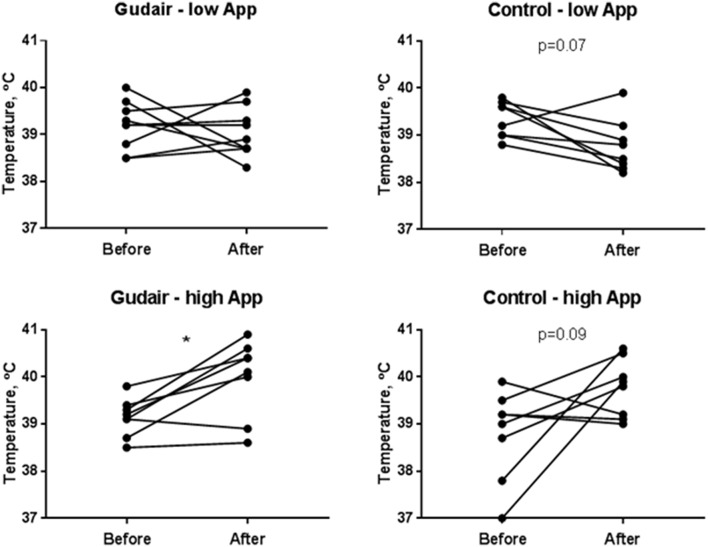
Rectal temperatures before and after challenge. Rectal temperatures immediately before challenge and the following day, comparing previously Gudair-vaccinated with control animals receiving a low dose (Gudair: *n* = 9; control: *n* = 8) or a high dose (Gudair: *n* = 8; control: *n* = 8) of *A. pleuropneumoniae* (App). Statistical analysis of change in temperatures after challenge using Wilcoxon matched-pairs signed-ranks test; *p < 0.05.

In the low *A. pleuropneumoniae* dose group, no behavioral changes could be observed during the 24 h following inoculation. In the high-dose group, several animals had an increased respiratory rate. On average, the rectal temperature slightly decreased following the low-dose challenge, particularly in the control group and only modestly so in the paratuberculosis vaccinated, whereas the temperature increased after the high-dose challenge irrespective of vaccination status, with no difference between paratuberculosis-vaccinated and control pigs 1 day after challenge (Figure 3). The weight change from day of challenge to the day after challenge was minor and not significant at any rate, although the miniscule trends corroborated the other clinical observations: Weight gain was smaller in animals receiving the high *A. pleuropneumoniae* dose vs. the low *A. pleuropneumoniae* dose. The weight gain was slightly negative in Gudair-vaccinated animals receiving the high *A. pleuropneumoniae* dose, but slightly positive in control animals (data not shown).

In the low *A. pleuropneumoniae* dose group, there were only minor lung lesions, and 9/17 had no lesions; there were no evident association between vaccination status and pathology score (Table 2). The high *A. pleuropneumoniae* dose induced lung lesions in all animals, and extensive reactions in 12/16 animals (pathology score 3) presenting with acute interstitial edema, acute lung necrosis (red hepatization) with fibrinogen exudation and fibrinous pleuritis. Of paratuberculosis vaccinated animals 8/8 had a pathology score of ≥2, whereas 5/8 in the control group scored ≥2 (*p* = 0.06). Overall, the pathology scores and the colony counts were positively correlated; 11/12 animals receiving the high-dose App with a pathology score of 3 also had a CFU >100.

**Table 2:** Colony counts and pathology scores.

	***n***	**Colony counts**					**Pathology score**			
		**0**	**1–10**	**11–50**	**51–100**	**>100**	**0**	**1**	**2**	**3**
**App high dose**, **~1** **×** **10**^**9**^ **CFU/animal (McFarland 5)**										
Gudair	9	9	0	0	0	0	4	5	0	0
control	8	5	0	1	0	2	5	1	2	0
						*p* = 0.05				*p* = 0.12
**App low dose**, **~1** **×** **10**^**8**^ **CFU/animal (McFarland 0.5)**										
Gudair	8	0	0	0	1	7	0	0	1	7
control	8	3	0	0	0	5	0	3	0	5
						*p* = 0.19				*p* = 0.06

*Colony counts after re-cultivation of lung tissues and pathological assessment of inner organs after challenge with A. pleuropneumoniae (App), comparing Gudair vaccinated with unvaccinated control animals. Statistical analysis using Kruskal-Wallis test for colony counts and Wilcoxon rank-sum test for pathology scores dichotomized as ≥2 vs. <2*.

After the low-dose challenge, *A. pleuropneumoniae* could not be isolated from lung tissues in any of the paratuberculosis vaccinated animals (0/9), but in 3/8 of the control animals (*p* = 0.05); in contrast, after the high *A. pleuropneumoniae* dose, bacteria were isolated in 8/8 paratuberculosis vaccinated, and in 5/8 control animals (*p* = 0.19) (Table 2). No bacteria were isolated from the liver, but *A. pleuropneumoniae* was isolated from the spleen in 1/8 paratuberculosis vaccinated (colony count of >100) and 1/8 control animal (colony count 51-100) after the high inoculation dose (data not shown).

With the very limited number of observations, there was no indication of a sex-differential effect of paratuberculosis vaccination on pathology score or bacterial burden of the lung tissues after the low *A. pleuropneumoniae* dose. After the high *A. pleuropneumoniae* dose, there was non-significant indication of an increase in pathology score and colony counts after paratuberculosis vaccination in females, with 4/4 paratuberculosis vaccinated female pigs having a pathology score of 3 contrasting only 1/3 control female pigs with a pathology score of 3 and 2/3 with a score of 0. No differences in distribution of pathology scores were observed in males across vaccination status (Supplementary Table 1).

**Figure 4:**
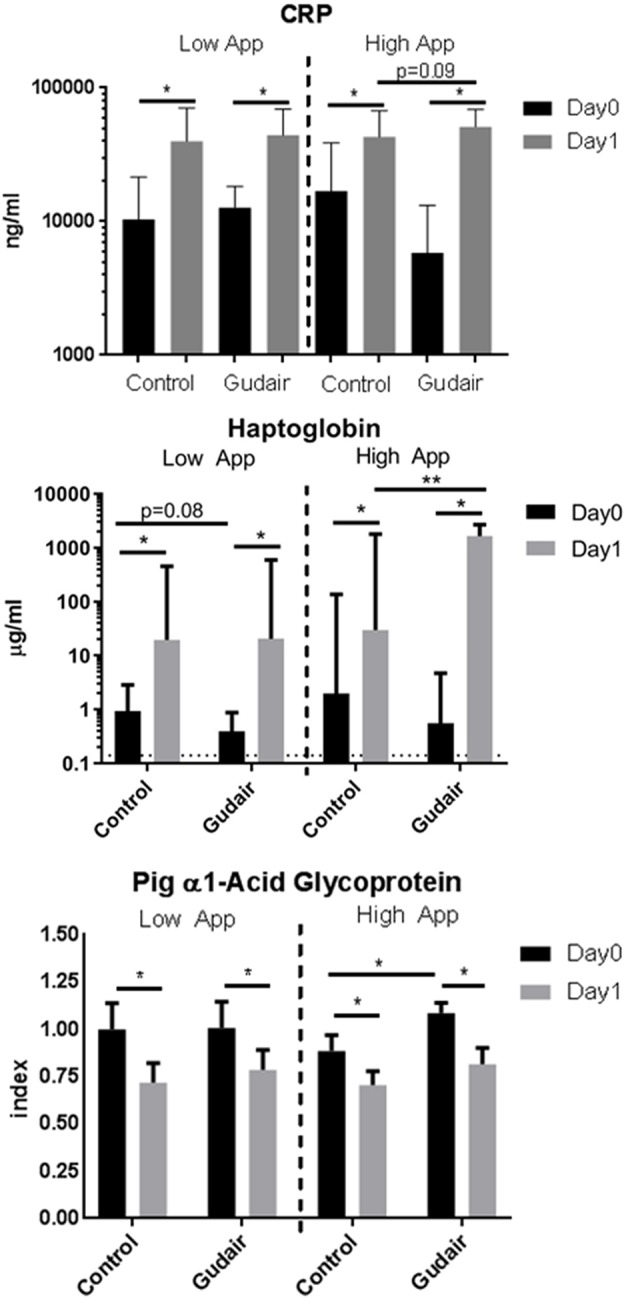
Acut-phase proteins before and after challenge. Means of concentrations of acute phase proteins C-reactive protein (CRP), haptoglobin and the negative acute phase protein pig α1-acid glycoprotein (PAGP) in serum immediately before challenge (day 0) and the following day (day 1), comparing previously Gudair-vaccinated with control animals; the animals were receiving a low dose (Gudair: *n* = 9; control: *n* = 8) or a high dose (Gudair: *n* = 8; control: *n* = 8) of *A. pleuropneumoniae* (App) on day 0. PAGP levels are relative to the index defined by the mean level of the low *A. pleuropneumoniae* control animals before challenge. Note the different scales in the sub-graphs. Statistical analysis of change in concentrations from day 0 to day 1 using Wilcoxon matched-pairs signed-rank test (for paired samples) and analysis of differences by Gudair vaccination on day 0 concentrations and on fold changes after challenge using Kruskal-Wallis test; *p < 0.05; **p < 0.01. Error bar is standard deviation. Hatched horizontal line on Haptoglobin graph is the lower limit of detection.

Before challenge, in pigs allocated for the high-dose challenge the serum levels of the positive acute phase proteins CRP and haptoglobin were slightly but non-significantly lower in paratuberculosis vaccinated pigs compared with control pigs, while the serum level of the negative acute phase protein PAGP was slightly higher (Figure 4). Other than that, there were no observed differences by vaccination status before challenge.

CRP and haptoglobin were increased after both low-dose and high-dose challenges in both paratuberculosis vaccinated and control pigs. The largest increase was seen in paratuberculosis vaccinated pigs receiving the high *A. pleuropneumoniae* dose. A similar inverse pattern was observed for the negative acute phase protein PAGP. For pigs challenged with the high *A. pleuropneumoniae* dose, this resulted in a significantly higher post-challenge haptoglobin level in paratuberculosis-vaccinated compared with control pigs (Figure 4).

Stratified by sex, although the sex-differential was not large or statistically significant in its own right, the effect of the paratuberculosis vaccine Gudair on the fold change of all acute phase proteins from before to after inoculation was larger in females compared with males, in line with the pattern observed for the pathology scores in the high *A. pleuropneumoniae* dose recipients (Supplementary Figure 2).

## Discussion

We found indication that Gudair vaccination exacerbated the pathology and systemic inflammatory response after inoculation with a high dose of *A. pleuropneumoniae* in young pigs, although a cautious interpretation is warranted due to the relatively small number of animals involved. In contrast, there was slight indication of an ameliorating effect of Gudair in animals receiving a low *A. pleuropneumoniae* dose challenge. There was no difference in leukocyte cytokine responses to polyclonal *ex vivo* stimulation in pigs vaccinated with Gudair compared with non-vaccinated controls.

The previously reported beneficial non-specific effects of Gudair have been suggested to be mediated via mechanisms of trained immunity (8, 9), as is indicated for BCG (6). The mycobacterium cell wall harbors several immunogenic constituents including muramyl dipeptide (MDP), the smallest peptidoglycan component of the mycobacterial cell wall and a ligand specific to NOD2, a cytoplasmic receptor of the innate immune system. MDP activation of NOD2 has been demonstrated to induce trained immunity of monocytes (6). MDP administration in mice prior to or immediately after a lethal challenge with *Klebsiella pneumoniae* reduced the mortality (25). Also a wide array of glycoconjugates interacts with the host immune system, including trehalose dimycolate (TDM), also known as mycobacterial cord factor, the major lipid in the outer membrane of mycobacteria. Pre-treatment with mycobacterially derived TDM has also been shown to improve survival of mice challenged with *K. pneumoniae* or *Listeria monocytogenes* (26).

Whereas, the animal studies discussed above find beneficial effects of inactivated mycobacterial vaccine formulations, some studies in humans find, however, that inactivation of vaccines abrogates the beneficial effects. A study in human adults found that inactivation of BCG may compromise the innate training effect. Compared with the live BCG, immunization with gamma-irradiated BCG induced only minimal effect on monocyte responses *ex vivo* to innate stimulation, albeit a significant increase in heterologous *ex vivo* immune (Th1/Th17) responses was found (27). Moreover, in an experimental sepsis study in human volunteers, immunization with gamma-irradiated BCG 5 days prior to i.v. administration of LPS did not ameliorate the endotoxemia-induced immunoparesis, measured as a decrease in *ex vivo* cytokine responses (28).

One explanation of this differential effect of live vs. killed BCG may be the shorter persistence of BCG in the killed formulation, and therefore a reduced exposure in time, immunological compartment space or dose of the innate training stimulants. An additional explanation may be that inactivation abrogates microbial RNA production and thereby greatly diminish the stimulation of the TLR8 pathway, which is important for monocyte activation and subsequent establishment of immunity including induction of T follicular helper cell differentiation, plasma cell maturation and humoral responses to vaccination (29).

In fact, the prevailing evidence from human clinical data and epidemiological studies find that beneficial non-specific effects are generally limited to live vaccines (30), including BCG (2), live attenuated measles vaccine (3, 31), oral polio vaccine (4) and smallpox vaccine (32); in contrast, inactivated human vaccines such as diphtheria-tetanus-pertussis vaccine have been associated with detrimental effects, particularly in girls (5). This dichotomy of live vs. inactivated vaccines was recently corroborated in an analysis of a large multicenter trial of a new malaria vaccine candidate, the non-live RTS,S vaccine (33). Here, despite a modest specific protection against malaria disease, the malaria vaccine was associated with a markedly higher mortality in females (34).

Observational studies in humans have indicated that the immunity transferred from the mother to the infant may enhance the beneficial non-specific effects of the BCG vaccine (35, 36) and the measles vaccine (37), presumably due to an interaction between the specific antibodies and the vaccine antigen in the recipient (38). Therefore, in order to take advantage of this potential enhancing effect of maternally derived immunity, all three sows received Gudair vaccine 3 weeks prior to farrowing. Whether the maternal immunization interacted with the vaccination of the offspring could not be elucidated under the present study design in which all offspring was born to immunized mothers. This hypothesis would be interesting to investigate in future studies. An interesting immunological feature in pigs is that in addition to uptake of maternally-derived antibodies, maternal leukocytes from the colostrum may also cross the intestinal barrier and enter lymphatic organs (39, 40).

To our knowledge there is no prior experience with the Gudair vaccine in swine, and the vaccine has not been tested in other animals in early life. We did not perform initial vaccine dose-optimisation studies in the piglets, but decided to apply half the standard dose recommended to goats and sheep above 6 weeks of age. The vaccine was immunogenic as evidenced by increased vaccine-specific interferon recall responses after vaccination, as well as reactogenic as evidenced by swollen regional lymph nodes, palpable nodules, and a few ulcers at the injection site.

In contrast to the few existing studies on non-mycobacterial effects of the mycobacterial veterinary vaccines, the present study was conducted in a vaccine-targeted pathogen-free setting, as the indoor-housed Danish industry pigs can safely be assumed to be free from paratuberculosis exposure.

The challenge organism used here is in the Gram-negative, facultative anaerobic coccobacillus *A. pleuropneumoniae* of the Pasteurellaceae family, which is a highly contagious pathogen endemic to modern farm pigs. Most commercially available vaccines against *A. pleuropnemoniae* are made from whole-cell inactivated bacteria and confer only partial and often serotype-specific protection (41). The *A. pleuropneumoniae* infection is associated with a local and systemic upregulation of pro-inflammatory cytokines and acute-phase proteins, in addition to widespread necrotic reactions in the airways directly mediated by the haemolytic and cytotoxic effects of bacterial LPS and Apx toxins or indirectly as a result of the provoked inflammatory host responses following the *A. pleuropneumoniae* induced damages (42). Whether mycobacterial vaccination non-specifically can enhance defense mechanisms against such endotoxin-mediated inflammation remains to be verified. As discussed above, there was no ameliorating effect of immunization with inactivated BCG 5 days prior to induction of experimental endotoxemia in human volunteers (28), nor were there any large effects of BCG vaccination on LPS *in vitro* stimulated cytokine responses in infants from Guinea-Bissau (43) or the UK (44).

Various non-adaptive defense mechanisms have been found of importance in clearance and/or amelioration of the immunological pathogenesis in *A. pleuropneumoniae* infections, including an effective mucociliary clearance mechanism which promote a rapid elimination of *A. pleuropneumoniae* (45); the ability of the host to exert micronutrient restriction of particularly iron; rapid recruitment of neutrophils and macrophages; effective opsonisation and killing of *A. pleuropneumoniae* by neutrophils and macrophages; complement-mediated bacteriolysis; tolerance to or neutralization of the potent bacterial toxins [reviewed in (42)]. Whether one or more of these mechanisms may be non-specifically enhanced by vaccination strategies, and in particular affected by precedent Gudair vaccination, remains to be investigated; the present study does not indicate a protective effect by a killed mycobacterial vaccine against heterologous infection.

Two different inoculation doses of *A. pleuropneumoniae* were applied. Prior experiments in our laboratory using the same strain of *A. pleuropnemoniae* (unpublished data) produced very little clinical and pathological effects; we therefore decided to split the infectious challenge in two parts separated by sufficient time to allow the evaluation and potential adjustment of optimal dose for the second half of the animals.

Whereas, the low dose gave only minor macroscopic necrotic lesions, the high dose produced severe acute lesions in all animals. This differential treatment obviously reduced the statistical power of our analysis, but in turn may have indicated an interesting differential effect of the immune activation on the subsequent challenge, with indication of an exacerbating effect of paratuberculosis vaccination in a high inoculation dose, contrasting the effect in the low inoculation dose.

In contrast to most of the murine challenge studies discussed above using survival as end-point and observing the animals over several days to weeks, we evaluated clinical, pathological and immunological end-points on sacrifice 24 h after challenge for animal welfare reasons due to the significant suffering imposed by the *A. pleuropneumoniae* infection.

The mode of administration of the inoculum bypassed the mucosa-associated lymphoid tissue barriers of the upper respiratory tract as the suspension of 2 ml bacteria was sprayed directly into the nostrils of the anesthetized animals and passively or actively inhaled to colonize the lower respiratory airways.

In addition, it was noted that the left lungs were consistently much more affected, as the sedated animals when returned to the pen after inoculation were laid to rest on the left flank. Hence, the inoculum had a somewhat focused non-natural distribution in the lungs.

Whereas, this may not fully mimic a natural way of exposure, leaving the possibility that immune checkpoints of the upper respiratory tract potentially affected by vaccination have been bypassed, the fact that *A. pleuropneumoniae* under natural conditions may be vectored on aerosol particles passing directly into the lower respiratory tract, where the bacteria preferentially bind to epithelial cells (46), renders the administration model relevant to evaluate the local defense property of the lower airways. Of note, a parenteral mode of i.v. or i.p. administration has typically been applied in the above discussed experimental septic murine models.

None of the experimental evidence of mycobacterial non-specific beneficial effects discussed above arises from studies in pigs. Although the pig is increasingly used as an experimental model organism, there are certain fundamental differences between the porcine and the murine or the human physiology, such as the higher body temperature of the pig, the inverted lymph nodes, higher gamma-delta T cell numbers in circulation, and differences in the epitheliochorial placentation prohibiting transplacental transfer of immunoglobulins (18, 47). Whether these or other biological differences may play an important role in non-specific immunity needs further investigation. Studies of BCG in human babies find that BCG has beneficial non-specific effects on mortality when given immediately after birth. Piglets were vaccinated within 2 days of life, at which stage the immune responses and functions are clearly dampened compared to later in life, but far from completely abrogated (48–50); one study found that an inactivated single-dose immunization against *Mycoplasma hyopneumoniae* of 4–5 day-old piglets significantly enhanced resistance to subsequent *M. hyopneumoniae* challenge (51). Herein, the increased specific adaptive *in vitro* IFN-γ responses in immunized pigs confirm the immunological competence of the young piglets to respond to vaccination.

## Conclusion

The inactivated Gudair vaccine against paratuberculosis marketed for use in ruminants did not protect young pigs against a subsequent heterologous challenge with a highly pathological dose of *A. pleuropneumoniae*, and in contrast may have had negative effects.

## Data Availability

The raw data supporting the conclusions of this manuscript will be made available by the authors, without undue reservation, to any qualified researcher.

## Ethics Statement

The animal experiments were approved by the Danish Animal Experiment Inspectorate, approval number 2015-15-0201-00520.

## Author Contributions

KJ, MH, PH, CB, and GJ designed the experiments. KJ and MH conducted the experiments. KJ analyzed the data. KJ drafted the first version of the manuscript. All authors contributed to the final version of the manuscript.

### Conflict of Interest Statement

The Gudair vaccines were kindly donated by CZ Veterinaria. CZ Veterinaria had no influence on the study design, data collection or analysis or writing of the present work. The authors declare that the research was conducted in the absence of any commercial or financial relationships that could be construed as a potential conflict of interest.

## Supplementary Material

Supplementary Figure 1 Example of local reactions to the Gudair vaccine, 0.5 ml. **(A)** Pig #86 33 days after vaccination, **(B)**: Necropsy 42 days after vaccination, presenting with severe pus exudate under the dermis; **(C)** In comparison, pig #52 with a milder local reaction at the injection site.

Supplementary Figure 2 Means of concentrations of acute phase proteins C-reactive protein (CRP), haptoglobin and the negative acute phase protein pig α1-acid glycoprotein (PAGP) in serum immediately before challenge (day 0) and the following day (day 1), comparing previously Gudair-vaccinated with control animals receiving a low dose of *A. pleuropneumoniae* (App) and stratified by sex or a high dose. PAGP levels are relative to the index defined by the mean level of the low *A. pleuropneumoniae* control animals before challenge. Error bars indicate standard deviations. Inserted table presents number of animals per treatment group and sex. Statistical test of difference in fold change of concentrations from day of App inoculation (day 0) to the day after inoculation (day 1) by vaccination status or sex, analyzed using Kruskal-Wallis; ^*^*p* < 0.05.

Supplementary Table 1 Pathology score of lungs and colony counts of Ap reseeded from lung tissues, same as in Table 2, stratified by sex.
