# Supplementary figures and images for: The Effect of Inactivated Mycobacterium Paratuberculosis Vaccine on the Response to a Heterologous Bacterial Challenge in Pigs
Source: Front Immunol. 2019 Jul 5;10:1557. doi: 10.3389/fimmu.2019.01557 (PMC6624675; doi:10.3389/fimmu.2019.01557)

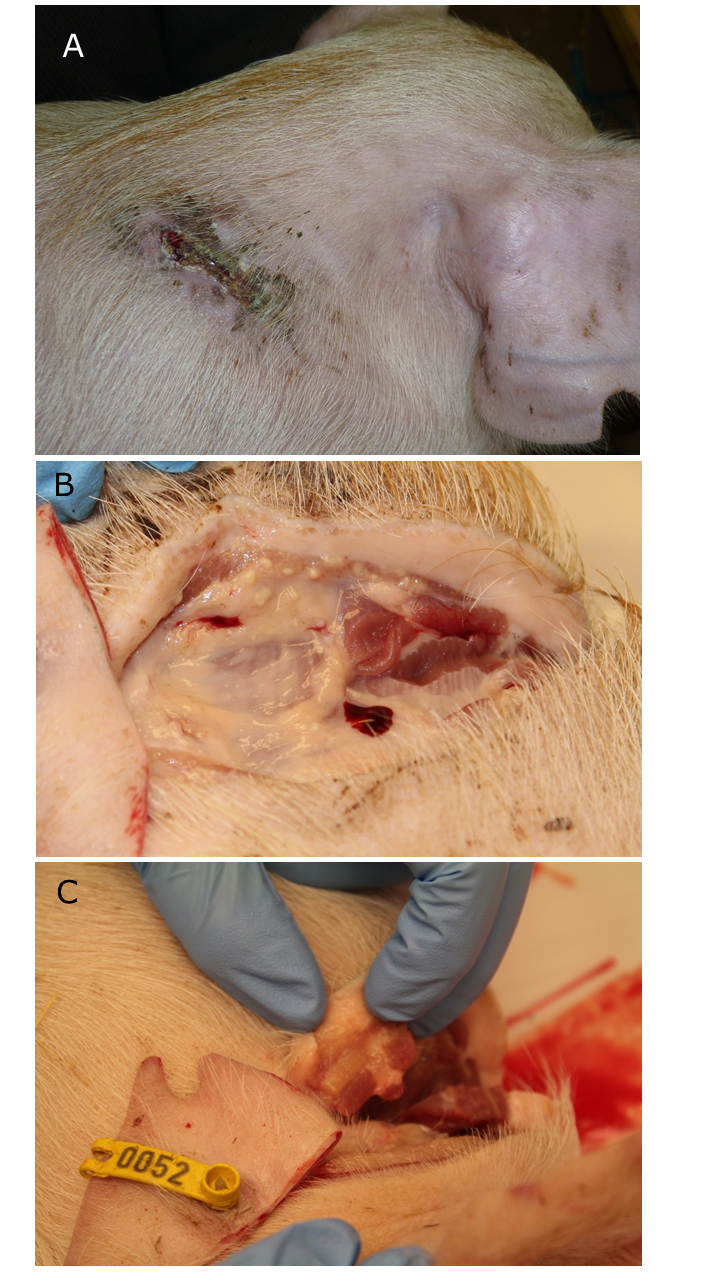

Supplement: Supplementary Figure 1 — Example of local reactions to the Gudair vaccine, 0.5 ml. (A) Pig #86 33 days after vaccination, (B): Necropsy 42 days after vaccination, presenting with severe pus exudate under the dermis; (C) In comparison, pig #52 with a milder local reaction at the injection site. [file Image_1.TIF]

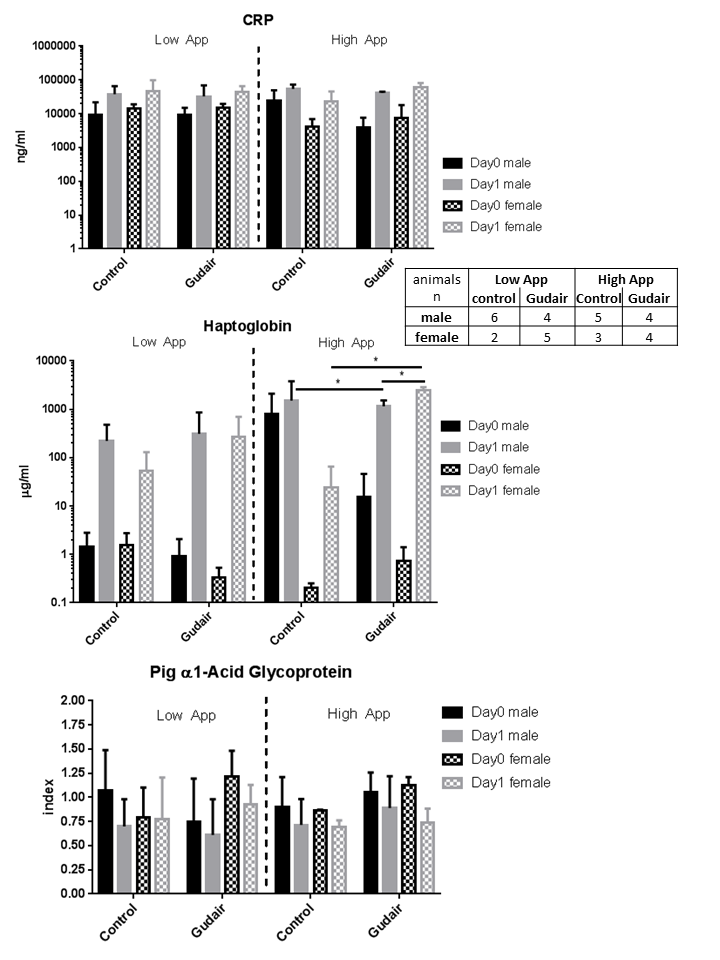

Supplement: Supplementary Figure 2 — Means of concentrations of acute phase proteins C-reactive protein (CRP), haptoglobin and the negative acute phase protein pig α1-acid glycoprotein (PAGP) in serum immediately before challenge (day 0) and the following day (day 1), comparing previously Gudair-vaccinated with control animals receiving a low dose of A. pleuropneumoniae (App) and stratified by sex or a high dose. PAGP levels are relative to the index defined by the mean level of the low A. pleuropneumoniae control animals before challenge. Error bars indicate standard deviations. Inserted table presents number of animals per treatment group and sex. Statistical test of difference in fold change of concentrations from day of App inoculation (day 0) to the day after inoculation (day 1) by vaccination status or sex, analyzed using Kruskal-Wallis; *p < 0.05. [file Image_2.TIF]
