# Supplementary material for: The Effect of Inactivated Mycobacterium Paratuberculosis Vaccine on the Response to a Heterologous Bacterial Challenge in Pigs
Source: Front Immunol. 2019 Jul 5;10:1557. doi: 10.3389/fimmu.2019.01557 (PMC6624675; doi:10.3389/fimmu.2019.01557)
Supplement: Supplementary Table 1 — Pathology score of lungs and colony counts of Ap reseeded from lung tissues, same as in Table 2, stratified by sex. [file Table_1.DOCX]

| **App dose** | **Vaccine** | **Sex** | **Colony counts** | | | | | **Pathology score** | |
| --- | --- | --- | --- | --- | --- | --- | --- | --- | --- |
|  |  |  | **0** | **1-10** | **11-50** | **51-100** | **>100** | **< 2** | **≥ 2** |
| Low dose | Gudair | M:  F: | 4  5 |  |  |  |  | 4  5 | 0  0 |
|  | control | M:  F: | 4  1 |  | 1 |  | 2 | 4  2 | 2  0 |
| High dose | Gudair | M:  F: |  |  | 1 | 4  3 |  | 0  0 | 4  4 |
|  | control | M:  F: | 1  2 |  |  | 4  1 |  | 1  2 | 4  1 |

Supplementary table 1. Pathology score of lungs and colony counts of Ap reseeded from lung tissues, same as in Table 2, stratified by sex. M=male; F=female. For the high App dose: The relative risk (RR) for having a pathology score ≥2 comparing Gudair vaccinated with controls: RR=1.25 in males, RR=3 in females. Mantel-Haenszel test of homogeneity in males vs females of the relative risk (RR), p=0.14. For low App dose, the statistical test of homogeneity in males vs females could not be performed due to multiple outcomes with zero observations.
